# A Case of Thrombosis of the Femoral Artery Leading to Gangrene

**Published:** 1893-06

**Authors:** Arthur E. Barker

**Affiliations:** Professor of Clinical Surgery at University College, and Surgeon to University College Hospital, London


### A CASE OF THROMBOSIS OF THE FEMORAL ARTERY LEADING TO GANGRENE.

BY

ARTHUR E. BARKER, F.R.C.S. Eng.,

*Professor of Clinical Surgery at University College, and Surgeon to University College Hospital, London.*

---

THIS patient was a man aged 55, though he looked older. This was probably due to the fact that for the last few years he had had very bad health, having suffered from frequent attacks of influenza, followed by extreme weakness. Besides this, he has been troubled with a stricture for some years past, and also with a urethro-rectal fistula, for which he was admitted to the Hospital on November 1st, 1892. His condition on admission was one of extreme debility, which he set down to the repeated attacks of influenza rather than to the consequences of the urinary affection. Beyond this, he did not look ill: his temperature was below 100°; his pulse fairly good; his urine was normal in appearance and acid in reaction; the heart-sounds were weak, but not otherwise affected.

From November 1st to Sunday, the 13th, he lay in bed, only getting up to go to the closet. He was fed on light, nourishing diet, and my house surgeon was instructed to pass a bougie occasionally for gradual dilatation of his stricture. On Sunday morning, the 13th, he was looking better than usual when I was in the ward, and told me in passing that he felt stronger. At noon the house surgeon passed a No. 7 French bougie without trouble; at 3.20 the patient had a rigor and a rise of temperature to 102.8°, falling soon to normal. At 11 p.m. he complained of a sudden and very severe pain in the whole of the left leg below the knee, and when my house surgeon was fetched he found the whole of the leg cold and blanched, and all sensation and motion abolished from the knee downwards. There was no pulsation in either the dorsalis pedis, posterior tibial, or popliteal arteries, and the femoral artery could only be felt to pulsate for about three inches below Poupart's ligament. In this condition he remained without much change up to 38 hours after the onset of the pain and loss of sensation and motion.

He did not then appear to be suffering at all. The lower part, including the foot, was pale, while the upper part of the skin was becoming purple up to a line encircling the knee at the level of the ligamentum patellæ. This purple discolouration extended beyond this line on the inner aspect of the knee in a patch about 3 inches long and 2 broad. The discolouration was sharply marked off against the skin, above which there was a slight flush. There was absolute loss of sensation and motion in all the structures below the knee, while there was great hyperæsthesia above the joint. There was no swelling or œdema anywhere; indeed, there was a slight shrinking of the leg as contrasted with its fellow. The femoral artery pulsated distinctly for about 3 inches below Poupart's ligament, but nowhere else could be felt any arterial pulse, which was in striking contrast to the opposite limb, where the arteries of the foot and thigh were well felt. His temporals were tortuous, as were also the radials to some extent; but none of these were fibrous to the finger, and nowhere could be felt anything clearly pointing to calcareous degeneration in the vessels. The superficial femoral on the affected side certainly felt hard and uneven, but scarcely to the extent of calcareous change. The heart sounds were normal, but feeble.

To what were the changes in the limb due? There can be no doubt that here was a case of thrombosis of the femoral artery in the upper part of its course. The mode of onset, the symptoms subjective and objective, were typical of this. It was a condition in its earliest stages, of which we often read but which fortunately we rarely see. This condition however deserves careful study, especially by operators, who live in dread of its supervention in cases in which they have tied the femoral artery high up for one reason or another. Here it had occurred spontaneously under our very eyes in a man not old in years, but old in tissue, and it offers an excellent opportunity for study.

Now what was the reason for this sudden blocking of the femoral artery? Was this a case of embolism or of thrombosis? After due consideration, I did not think there was evidence of an embolic blockage, although the suddenness of the attack suggested it. At all events, we could exclude those emboli which are found to start from vegetations on the cardiac valves as the result of acute or chronic endocarditis. There was no abnormality discoverable about the valves of the heart, and the temperature before and after the attack negatived acute endocarditis. Of course a clot may have formed on some atheromatous patch in the aorta, and from this may have been dislodged and swept on into the femoral. But the most reasonable explanation seems to be that a thrombus had formed on the spot in the femoral. What we could feel of the vessel was distinctly hard and knotty, and we know that this particular artery is prone to changes due to chronic arteritis. It is easy then to suppose that some slight inequality on the inner surface, such as the edge of a calcareous plate or an atheromatous ulcer (so called), may have been the starting point of a clot which ultimately blocked the lumen of the vessel. Had the condition which led to the sudden rise of temperature and rigor anything to say to this clotting? I cannot help thinking that it had. The sudden fever to my mind suggested an influx of septic matter to the blood from one source or another. Possibly this was due to disturbance of some septic focus in the urethra by the bougie. It is at all events significant that this rigor took place three hours after the use of the bougie, and the thrombosis started a few hours later. Now we know that in septic conditions clots are frequently formed in the blood stream: for example, in embolic pyæmia and in acute endocarditis. And I suppose that some analogous septic blood change was produced in this case, and that the altered blood only required a roughened surface to provoke it to coagulate, so to speak. It certainly was not ordinary pyæmia, for the temperature had been quiet both before and after the attack. But a much less profound blood change than that produced in the latter disease might, I apprehend, lead to the formation of thrombi in a very debilitated man with diseased arteries.

The changes produced in the leg, as the result of the arrest of its blood supply, were clear enough. The total anæmia of the nerves resulted in arrest of their powers and consequent loss of both sensation and motion. The sudden onset of pain at the beginning of the attack was possibly due to sudden and general contraction of all the vessels of the limb when their blood supply was cut off, and has its analogue in the pain produced by the application of extreme cold to a part. The pallor is explained in the same way, and also the loss of heat. We also see clearly the commencement of mummification as indicated by the shrinking and dryness of the whole limb, so different from the moist gangrene due to injury to the main vein.

As to the treatment of the condition, but very little remedial could be done. In some cases it has been suggested to break up the clot by manipulation, so that it might be driven on piecemeal into smaller vessels where it would not produce such serious consequences. But this is an expedient which could rarely be adopted with any success. But failing this, we must in all such cases aim at keeping up the warmth of the limb by wrapping it in cotton wool and placing hot bottles in the bed. In milder cases where there is a chance of the collateral circulation establishing itself, this maintenance of an equable temperature will greatly assist nature in restoring the circulation, and the same may be said of rest and light nourishing diet. Opium may be required to allay pain; but alcohol should, if possible, be avoided at the onset, lest it excite the circulation overmuch.

The prognosis was of course very grave. There appeared to be no sign of collateral circulation, while there was every sign that the limb from the knee downwards was mortifying. The upper border of the purple area was becoming suspiciously defined, while the foot was becoming more and more sallow and greyish. In a patient so very much debilitated as this man was to begin with, all this aroused serious forebodings, and even the prospect of relieving him by amputation later on was not a good one.

The sequel of this case was, however, more favourable than might have been expected. The gangrene progressed steadily until the whole leg up to some inches above the knee was becoming putrid. Then a large abscess in the lower part of the thigh slowly formed until the limb was a bag of pus. At the same time herpes appeared on the face, nose, and lip, and there was troublesome cough. The general condition, however, improved in a measure. The pulse came down to 100, and also gained in quality. The temperature ranged from normal to 100° and 101°, and he took nourishment well. On December 2nd, I consequently felt justified in removing the gangrenous limb, and accordingly amputated by the modified circular method in the middle of the thigh. Part of the abscess reached up into the stump left after division of the femur 8½ inches above its lower end. Below this all the muscles were rotting and bathed in pus. The wound was flushed with hot water, and the abscess track in the stump scraped clean. No ligatures were required, as the arteries were all blocked. The flaps were stitched closely together, a large drain tube being placed across the stump. A salicylic wool dressing and firm bandaging completed the operation.

In the parts removed the vessels showed no disease, but were blocked by firm dark clot.

Recovery was slow, but steady and uneventful, and the patient left the hospital on January 25th, 1893, with a sound but somewhat conical stump. He has written to me this month (May, 1893), and only complains now of the same great debility from which he suffered before.

I find two other similar cases of thrombosis of arteries during influenza recorded in the *Centralblatt für Chirurgie* of April 1st, 1893. In one, gangrene of the leg was the consequence, and amputation had to be performed, recovery following. In the other, the brachial artery was thrombosed, but no gangrene occurred.

